# *BRAF* V600E Mutation Has Variable Tumor-Specific Effects on Expression of MAPK Pathway Genes That Could Affect Patient Outcome

**DOI:** 10.3390/ijms26167910

**Published:** 2025-08-16

**Authors:** Sourat Darabi, Phillip Stafford, David R. Braxton, Carlos E. Zuazo, Taylor J. Brodie, Michael J. Demeure

**Affiliations:** 1 Hoag Family Cancer Institute, Newport Beach, CA 92663, USA; phillip.stafford@asu.edu (P.S.); david.braxton@hoag.org (D.R.B.); taylor.brodie@hoag.org (T.J.B.); michael.demeure@hoag.org (M.J.D.); 2 School of Life Sciences, Arizona State University, Tempe, AZ 85287, USA; 3 Fulgent Genetics, El Monte, CA 91731, USA; 4 Translational Genomics Research Institute, Phoenix, AZ 85004, USA

**Keywords:** *BRAF* mutations, RNA expression, whole exome sequencing, whole transcriptome sequencing, thyroid cancer, melanoma

## Abstract

BRAF inhibitors have a 50–70% response rate in melanoma but are less effective for thyroid cancer. Differential response may be from activation or expression of downstream mitogen-activated protein kinase (MAPK) pathway genes. Retrospective analysis compared whole exome and transcriptome sequencing in melanoma and thyroid cancers from April 2019 to October 2023. The MAPK Activation Score (MPAS) was calculated using Z-score normalized/log-transformed values indicating expression across 10 MAPK-associated genes. Our tumor registry provided outcome data. *BRAF* V600E mutations were identified in 33 of 200 (17%) melanomas and 14 (7%) had other *BRAF* mutations (V600K/R). Of 49 thyroid tumor samples, *BRAF* V600E mutations were found in 19 (39%). RNA expression of BRAF and the 10 MAPK-associated genes were increased in melanomas with V600E compared to wild-type *BRAF* (*p* = 0.02). Conversely, *BRAF* V600E mutation in thyroid cancer was not associated with increased expression nor MAPK pathway activation. No significant difference in overall survival based on *BRAF* mutation was observed in the subset of patients where data was available. The MAPK pathway is differentially affected by the different cancers, with increased MAPK activation observed in melanoma and not in thyroid cancer. This may account in part for the observed differential response to BRAF inhibitors.

## 1. Introduction

Genomic databases offer an opportunity for investigators to study biologically interesting questions. The insights garnered may be more powerful when the genomic data is annotated with patient clinical data and outcomes of treatment. The cBioPortal for Cancer Genomics is an open-access, open-source resource that provides visualization, interrogation, analysis, and download of large-scale cancer genomics data sets [1,2]. To this end, our hospital has developed its own instance of cBioPortal to archive its genomic data from several clinical commercial labs, and we are in the process of integrating this data with our tumor registry database. To date, our cancer center has collected genomic data from nearly 6000 independent patient tumors. The biologically relevant question we address herein is why there may be a differential response to BRAF inhibitors between two cancers in which a *BRAF* V600E mutation is the most frequent genomic driver observed. This mutation results in the substitution of a valine (V) with a glutamic acid (E) amino acid at position 600 in the BRAF protein, causing constitutive activation of the mitogen-activated protein kinase (MAPK) pathway [3]. This pathogenic *BRAF* V600E mutation is an oncogenic driver in many melanomas, colon, thyroid and NSCLC cancers and is a drug target for BRAF inhibitors including vemurafenib, dabrafenib and encorafenib [4].

An activating mutation in *BRAF* occurs in over 50% of malignant melanomas, most of which are *BRAF* V600E [5]. BRAF inhibitors in *BRAF* mutant melanoma have a response rate of approximately 50% to 70% [6,7]. *BRAF* V600E mutations also occur in 40–60% of thyroid cancers [8,9]. The response rate to BRAF inhibitors in advanced thyroid cancer, however, is only 29—38.5% in *BRAF* mutant thyroid cancer, a substantially lower rate than is seen with melanoma [10,11]. The response rates could differ due to several mechanisms. There could be a differential expression of the mutated gene or decreased expression of downstream gene sets. Alternatively, the upregulation of resistance pathway genes may diminish the efficacy of BRAF targeted inhibitors. We were able to explore each of these hypotheses using the genomic database we established. Accordingly, we compared tumor transcriptomes to assess the activation state of the MAPK pathway and resistance pathways in our melanoma and thyroid cancer patients.

## 2. Results

A retrospective analysis using our internal cBioPortal database from April 2019 to October 2023 showed that of 200 melanoma samples, a *BRAF* V600E mutation was identified in 33 (17%) samples. The rate in our data was consistent with reported percentages of *BRAF* V600E in melanomas seen in The Cancer Genome Atlas (TCGA) and AACR Genomics Evidence Neoplasia Information Exchange (GENIE) databases (35% [*n* = 480] and 21% [*n* = 7915], respectively) [1,2]. Furthermore, in our cohort, 14 of the 200 (7%) melanoma samples had other *BRAF* mutations, including *BRAF* V600K (*n* = 13, 26%) and *BRAF* V600R (*n* = 1), and 19 (9.5%) had other miscellaneous *BRAF* mutations, such as *BRAF* D594N (Table 1). Therefore, in all, we had a total of 33% of melanoma patients with a *BRAF* mutation. The demographic data of the patients included in our analysis are detailed in Table 2 and Table 3. *BRAF* V600E mutations were identified in 19 (39%) of the 49 thyroid tumor samples in our database. The prevalence of *BRAF* V600E mutations in thyroid cancers in TCGA and AACR GENIE databases were (59% [*n* = 500] and 41% [*n* = 2769], respectively). TCGA is skewed toward resected surgical samples, possibly accounting for a higher rate of mutation. We did not find any other pathogenic *BRAF* alterations in our thyroid cancers.

**Figure 1:**
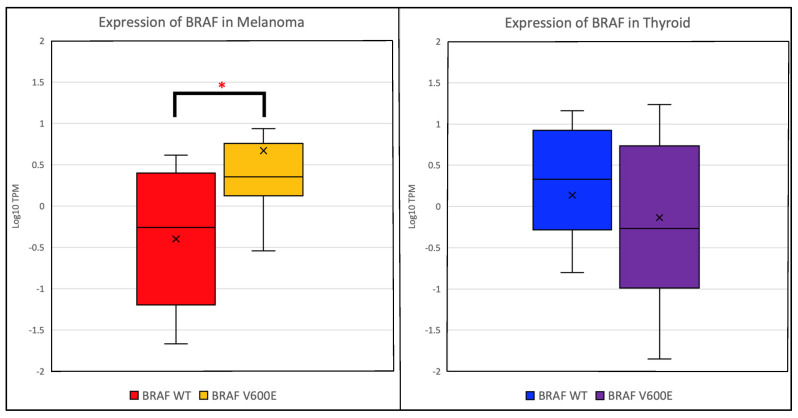

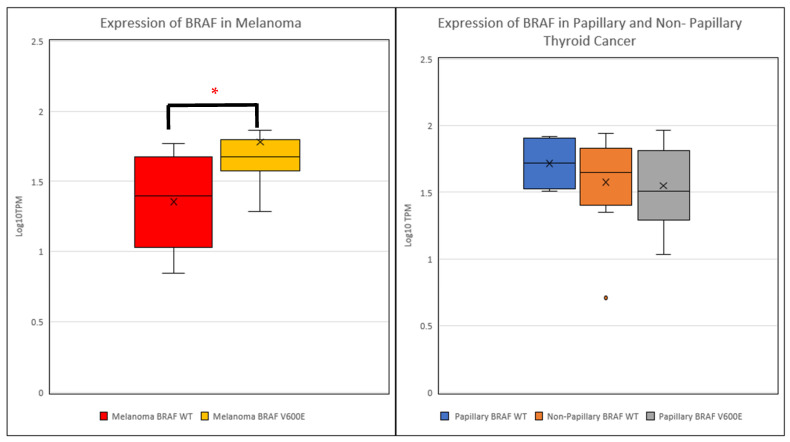
Boxplot of expression of *BRAF* in melanoma (left) and papillary vs. non-papillary thyroid (right). In *BRAF* V600E cases, the expression profile of that cohort is plotted next to a cohort where *BRAF* was WT. In melanoma, *BRAF* is more highly expressed in patients harboring the V600E mutation than in patients with WT *BRAF* (significant at *p* = 0.001) in the above figure statistical significance is represented by an asterisk, while in thyroid cancer (papillary *BRAF* WT, non-papillary *BRAF* WT, and papillary *BRAF* V600E), the expression of *BRAF* is higher when *BRAF* is WT (*p* = 0.134).

**Figure 2:**
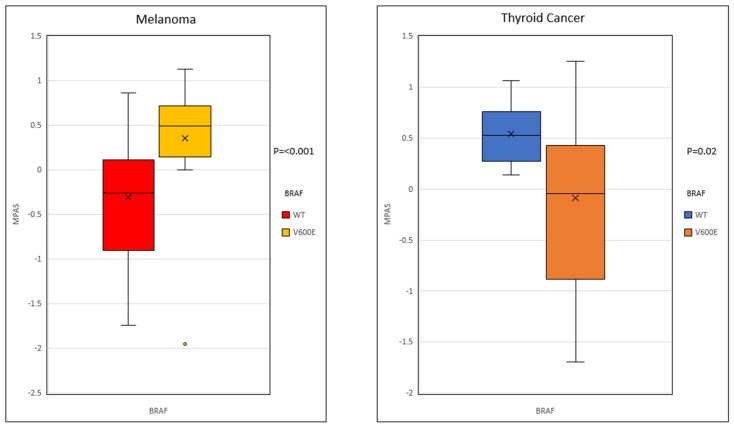

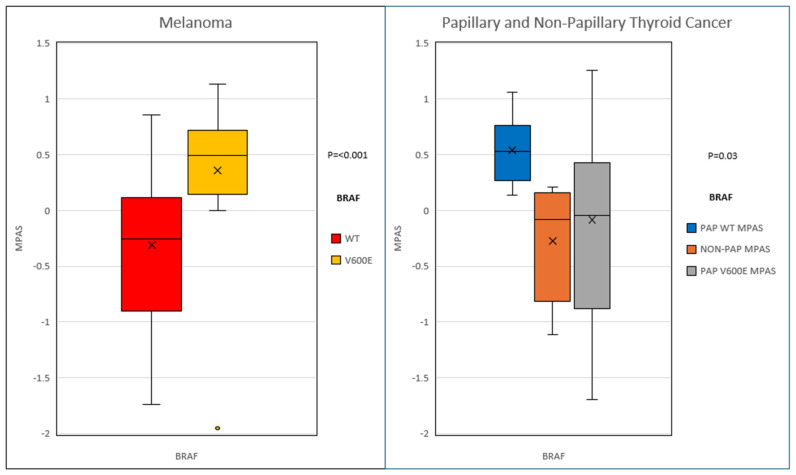
The trend of expression of these genes in papillary thyroid, non-papillary thyroid and melanoma. The *p*-value between patients with *BRAF* V600E and those with WT *BRAF* is *p* = <0.001 and *p* = 0.03 for papillary thyroid cancer. For thyroid the differences were less profound; no pairwise comparison was significant.

We found that mutant *BRAF* is overexpressed in melanoma but not thyroid cancer (Figure 1). Overall expression of mutant *BRAF* in melanoma was mean 141.61 ± 268.03 transcripts per million (TPM) (mean ± standard deviation) compared to 28.31 ± 17.9 in WT BRAF (*p* < 0.005). The *BRAF* expression in patients with papillary thyroid cancer with the *BRAF* V600E variant of 43.2 ± 25.72 was similar to that seen in WT (55.8 ± 22.10 TPM) (*p* = 0.3). Furthermore, we further observed an increased MAPK activation in melanomas harboring V600E mutations when compared to WT (median MPASs: 0.49 vs. −0.25, respectively, *p* = 0.02). Conversely, *BRAF* V600E mutation in thyroid cancer did not show a marked difference in MAPK pathway activation compared to WT (median MPASs: = 0.05 and −0.53, respectively, *p* = 0.59) (Figure 2).

**Figure 3:**
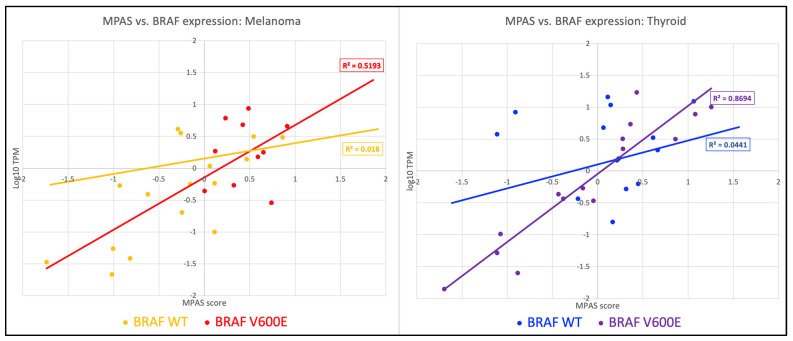

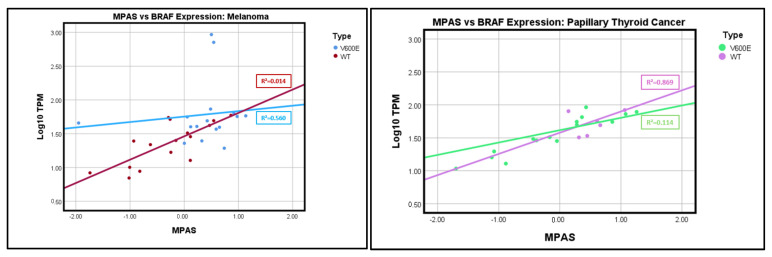
MPASs are plotted against the expression values (displayed as log_10_) of *BRAF* for both wild-type and V600E. A general trend exists between the MPASs and *BRAF* expression in Papillary Thyroid cancer in V600E patients (R^2^ = 0.87), while in Melanoma the same trend remains but is less obvious (R^2^ = 0.014).

In both melanoma and papillary thyroid cancer, however, expression of *BRAF* did correlate with MAPK activation as reflected by the MPAS (Figure 3). The correlation of MPAS with the expression of *BRAF* RNA in patients with thyroid cancer suggests that MAPK activation in papillary thyroid cancer may be dependent on the degree of expression of the *BRAF* oncogene. This relationship was not seen to such a strong degree in melanoma. It is possible that high MPAS activation dependent on increased *BRAF* expression could reflect tumors that might be more sensitive to BRAF inhibition [12], but we could not evaluate this hypothesis using our database.

**Figure 4:**
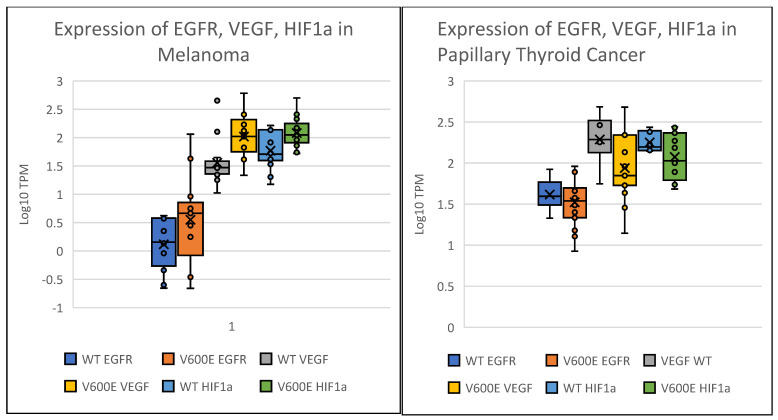
The Boxplots show the expression in TPM of *EGFR*, *VEGF* and *HIF1A* in both papillary thyroid cancer and melanoma. Expression of potential targets of MAP kinase pathways in Melanoma (left) and papillary Thyroid (right). We examined the expression of select growth factors that might be impacted by MAP kinase activity. The log_10_ expression values in TPM for *EGFR*, *VEGF*, *HIF1a* are shown. For melanoma the difference between expressions of *HIF1a* is significant (*p* = 0.006) and *VEGF* (*p* = 0.015). In papillary thyroid the differences in expression are not significant.

**Figure 5:**
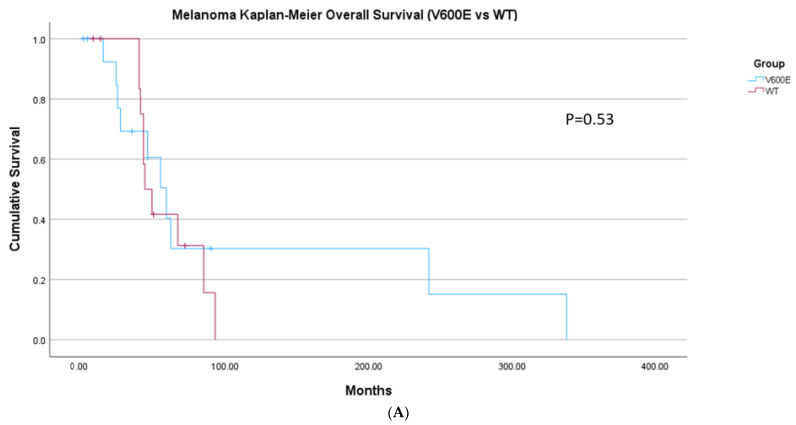

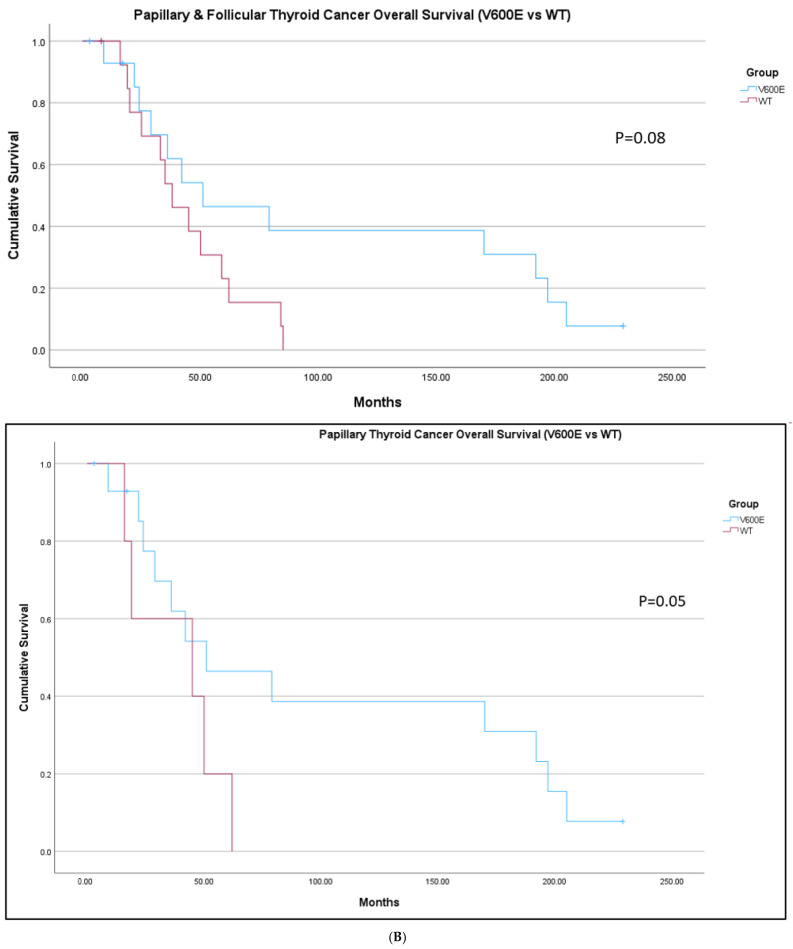
(**A**) Overall survival in a subset of patients with Melanoma with and without *BRAF* V600E mutation. (15 patients with V600E vs. 14 WT). (**B**) Overall survival in a subset of patients with papillary Thyroid Cancer 15 patients with V600E 6 WT filtered to include only those papillary morphology).

A lack of response to BRAF inhibitors could also be due to overexpression of resistance pathways, notably *EGFR* as is seen in colon cancer [13]. We compared the expressions of *EGFR*, *VEGFA*, and *HIF1A* in a subset of patients that included the 30 patients with thyroid cancer and 32 patients with melanoma for whom expression data was available. *EGFR* expression in melanoma ranges from 0.8 to 1.75, which falls within the expected range of *EGFR* expression in melanoma tumor cells. However, in melanoma cells that harbor a *BRAF* V600E mutation, expression of *EGFR* ranges from a low of 0.8 up to 3.1 which is well above the expected expression levels of *EGFR*. The same trend holds for *VEGF* and *HIF1a*, where these genes are expressed with the expected range of values compared to historical melanoma cases with WT *BRAF*, but significantly higher in melanoma with *BRAF* V600E genotype. In papillary thyroid cancer, *EGFR* expression has a larger range of expression values than in melanoma, with no significant difference in expression between WT and V600E *BRAF* tumors. This holds true for *VEGF* and *HIF1A* as well—in thyroid there is no significant difference between the average TMP expression values for thyroid cancers with *BRAF* WT when compared to *BRAF* V600E tumors (Figure 4). Additionally, we were able to obtain survival data for the patients in our cohort through our cancer data services. We found no significant difference in overall survival was observed between patients with *BRAF* V600E and WT in melanoma (*p* = 0.5 Figure 5A). The survival in papillary thyroid cancer cohorts compared to WT (*p* = 0.05) (Figure 5B). For thyroid cancer, we limited our OS cohort to papillary and follicular cancers for uniformity and to exclude cancers with notably worse prognosis, such as medullary thyroid or anaplastic thyroid cancer.

## 3. Discussion

The adoption of DNA sequencing technologies into routine clinical care of patients with cancer has been increasingly driven by the expanding availability of a diverse array of targeted therapies. The development of BRAF inhibitors has been a notable advance in the treatment of various cancers.

*BRAF* is part of the MAPK/ERK signaling pathway, which regulates growth and cell division. Mutations in *BRAF*, most commonly V600E, can lead to uncontrolled cell proliferation by constitutive activation and continued signaling for cell growth. *BRAF* V600K, V600E, and V600R all have slightly different impacts on survival [14]. Approximately, 50% of melanomas carry *BRAF* mutations (90% are V600E) [15]. About 50% of papillary thyroid cancers and 24.1% of follicular thyroid cancers harbor a *BRAF* V600E mutation [16]. *BRAF* is a serine-threonine kinase member of RAF family that has a critical role in the mitogen-activated protein kinase (MAPK) pathway. The most common mutations in codon 600 lead to dysregulation of kinase activity and activating of MAPK pathway, although studies have shown that some non- V600 mutations also lead to MAPK activation [17]. MAPK pathway is involved in several molecular and cellular processes such as cell division, apoptosis, and cell differentiation [18].

In 2022, the FDA gave approval for the treatment of solid tumors with mutant *BRAF* using dabrafenib, a BRAF inhibitor, and trametinib, a MEK inhibitor [19]. Initially, the approval was for *BRAF* V600E mutations for only a few cancers such as melanoma, non-small cell lung cancer, and anaplastic thyroid cancer but evidence of added efficacy in other cancers led to pan-tumor approval. These additional studies including the BRF117019 and NCI-MATCH trials showed responses in patients with high-grade gliomas (HGG), biliary tract cancer, low-grade gliomas (LGG), hairy cell leukemias, adenocarcinomas of the small intestine, gastrointestinal stromal tumors, as well as anaplastic thyroid cancers [20]. The response rates reported vary from 12% in colon cancer to 89% for hairy cell leukemia [21]. The variable response rate is not clearly understood but we conjecture that it could be context dependent. In certain tumors, allele-specific expression of the *BRAF* V600E mutant allele could be dominant or blunted. One could have, alternatively, over-expression of resistance pathways as is seen with activation of the EGF pathway [22]. In colon cancer, for example, a *BRAF* V600E mutation in approximately 10% of patients is associated with a poor prognosis [23]. In colon cancer cell lines harboring *BRAF* V600E mutations, *EGFR* re-activates MAPK conferring resistance to BRAF inhibition with vemurafenib [24]. In papillary thyroid cancer, a *BRAF* mutation occurs in 36–83% of cases [25] and, although of some debate, by itself *BRAF* mutation does not likely confer an adverse prognosis. Both dabrafenib and vemurafenib have been evaluated as single-agent BRAF inhibitors in patients with advanced, radioiodine refractory papillary thyroid cancer. Dabrafenib yielded a 29% response rate of 29% and stable disease in 45%. Vemurafenib was associated with a 38.5% response rate albeit with increased toxicity [11]. In 2011, melanoma was the initial approved indication for vemurafenib based on improved overall survival compared to that offered by other approved treatments. The response rate seen in this study of 675 treatment naïve patients was 48.4% [26].

MEK inhibitors such as trametinib and cobimetinib target MEK1 and MEK2, proteins in the MAPK signaling pathway. Cobimetinib binds to MEK1 and MEK2, inhibiting their activity and preventing the phosphorylation and activation of ERK, downstream of the MAPK pathway. When used in combination with an anti-BRAF drug like vemurafenib, signaling escape of the BRAF pathway is inhibited.

Several studies measured transcription of the *BRAF* gene when the V600E mutation is present. The mutation likely has little direct effect on the expression of the *BRAF* gene, the rate of the V600E transcript degradation, or recycling rate. It does affect protein function, but we would expect generally similar mRNA copy numbers of *BRAF* WT and *BRAF* V600E [27]. It is possible, however, that mutant *BRAF* could be differentially expressed. We examined gene expression and BRAF activation using the MPAS, which is a metric that provides insight into the aggressiveness of tumor cell growth and proliferation. These kinase inhibitors can impact the MAPK/ERK pathway but escape signaling in turn can alter gene expression of negative feedback loops from crosstalk pathways like PIK3/AKT and mTOR. We examined the expression of 3 genes to interrogate whether increased expression of *EGFR*, *VEGF*, or *HIF1a* might indicate activation of escape resistance (Figure 4). In melanoma but not thyroid cancer, *BRAF* V600E mutation is associated with increased expression of *VEGF* and *HIF1a.* Taken together, the results shown here indicate that although the *BRAF* V600E mutation is present in both thyroid and melanoma cases, the link between mutation and the MPAS and *BRAF* expression are quite different. This observation suggests that treatment of papillary thyroid cancer and melanoma by targeting BRAF and MAPK/ERK pathways should be studied in the context of the different regulatory mechanisms by which *BRAF* is up or downregulated in these two distinct cancers.

Our work does have some limitations. Most notably, our findings should be viewed as hypothesis generating and further investigations are needed for functional confirmation. Our clinical data is retrospective, and there is incomplete annotation, particularly regarding treatment outcomes for some of the patients. We were unable to look at allele specific expression as a potential variable. Ideally, transcriptomic data would be normalized to adjacent normal tissue; however, we feel that since RNA was assessed similarly in the two tumor types and despite *BRAF* mutational status, comparisons between groups are valid. We do not have proteomic data looking at phosphorylation states of kinase pathway proteins, which could be performed using a reversed phase protein array and would be a worthwhile further investigation in our lab [28]. Our data and conclusions are descriptive and can lead to other potential further experiments for validation such as RT-PCR. Such in vitro testing should be performed to validate the concepts expressed here. In conclusion, our report demonstrates that an integrated multimodal clinical and genomic database can be informative and lead to the generation of hypotheses guiding investigations elucidating important oncogenic processes.

## 4. Methods and Materials

Our hospital maintains a database of genomic data from a variety of patient tumors sequenced for clinical care in our private instance of cBioPortal. This password-protected secure database is automatically updated on a daily basis as we receive genomic reports. The cBioPortal is maintained by a third-party vendor (The Hyve, Utrecht, The Netherlands). The data is currently uploaded by the clinical laboratory that provides genomic testing to our patients. As of 09/30/24, we have 5881 samples in our database.

This study cohort included patients from our institution who were diagnosed with melanoma or thyroid cancer from April 2019 to October 2023. Genomic data is obtained from the results of clinical sequencing performed for patient care through commercial labs. Formalin-fixed paraffin-embedded (FFPE) samples from patients with cancer were submitted to a commercial CLIA-certified laboratory for molecular profiling (Caris Life Sciences, Phoenix, AZ, USA). This study follows guidelines provided by the Declaration of Helsinki, Belmont Report, and USA. Common Rule. In accordance with compliance policy 45 CFR 46.101 (b). Because this study was conducted using retrospective, de-identified clinical data, patient consent was not required, and the study was considered IRB-exempt.

### 4.1. DNA Next-Generation Sequencing (NGS)

Patient samples were provided to Caris Life Sciences as slides. Pathology uses one slide for H&E and in some cases additional slides are used for immunohistochemistry (IHC) but the remainder are tumor enriched by harvesting targeted tissues using manual microdissection techniques. Genomic DNA was extracted from FFPE tissue samples and subjected to NGS using the NovaSeq 6000 Sequencer (Illumina, Inc. San Diego, CA, USA). Hybrid capture uses a custom SureSelect Whole Exome V7 capture panel (Agilent Technologies, Santa Clara, CA, USA) which boosts the capture of 719 cancer-related genes to obtain >1000× average coverage, with at least 500× coverage for the remaining genes. All variants were detected with >99% confidence based on allele frequency and amplicon coverage, with an average sequencing depth of coverage >500× with a sensitivity down 5% VAF. Critical variants with sufficient coverage (10 unique variant reads with at least 20% tumor and 100 total allele reads) are reported even if below the guaranteed 5% VAF threshold. (https://www.carislifesciences.com/physicians/physician-tests/mi-tumor-seek-hybrid/ (accessed on 1 September 2024)). Certified molecular geneticists examined the identified genomic variants and categorized them in alignment with the standards set by the American College of Medical Genetics and Genomics (ACMG). Calculation of mutation frequencies in individual genes included “pathogenic” and “likely pathogenic” variants, while those labeled as “benign”, “likely benign”, and “variants of unknown significance” were excluded.

### 4.2. Whole Transcriptomic Sequencing

Formalin fixed paraffin-embedded (FFPE) specimens underwent pathology review to measure percent tumor content and tumor size; a minimum of 10% of tumor content in the area for microdissection was required to enable enrichment and extraction of tumor-specific RNA. The Qiagen RNA FFPE tissue extraction kit was used for extraction, and the RNA quality and quantity were determined using the Agilent TapeStation. Biotinylated RNA baits were hybridized to the synthesized and purified cDNA targets and the bait–target complexes were amplified in a post capture PCR reaction. The Illumina NovaSeq 6000 was used to sequence the whole transcriptome from patients to an average of 60M reads. Raw data was demultiplexed by Illumina Dragen BioIT accelerator, trimmed, counted, PCR-duplicates removed and aligned to human reference genome hg19 by STAR aligner. For transcription counting, transcripts per million values were generated using the Salmon expression pipeline. The pipeline does retain sequence information about the mutations in the RNA, but the expression values used in this manuscript reflect all transcripts of the gene being described. Due to the effects of allelic dominance, methylation, and other epigenetic factors, when describing patients with a V600E mutation in *BRAF*, we cannot assume that all *BRAF* transcripts express the V600E mutation [29].

### 4.3. Data Analysis

Post-sequencing analysis included correlation coefficient, estimation of dynamic range across samples and a percentile calculation of expression values within cancer types. The MAPK Pathway Activation Score (MPAS) was calculated as the average z-score of expression values (in TPM units) of 10 MAPK-associated genes (*PHLDA1*, *SPRY2*, *SPRY4*, *DUSP4*, *DUSP6*, *CCND1*, *EPHA2*, *EPHA4*, *ETV4*, and *ETV5*) as described by Wagle et al. [12]. MPAS serves as a transcriptomic measure of the activation state of the MAPK and a biomarker for *KRAS* and *BRAF* mediated tumor proliferation. We compared the MPAS between *BRAF* V600E versus *BRAF* WT in both melanoma and thyroid cancers. We analyzed available cases per tumor type, 15 patients with *BRAF* V600E mutations compared to 15 *BRAF* WT tumors in thyroid cancer (6 papillary WT, 8 non-papillary WT, and 15 papillary V600E) and 16 V600E vs. 16 WT cases in melanoma. Although there were many melanoma and thyroid patient samples, we selected samples where data was consistent, was of sufficient quality, was run under the same capture set, and had transcriptome data for all genes.

Additionally, we evaluated overall survival (OS) in a subset of patients the data was available through our cancer data services (cancer registry). We were able to evaluate OS in 21 patients with papillary thyroid cancer histology and 29 patients with melanoma.

In order to interrogate the possible role of resistance mechanisms, we examined the RNA expression of *EGFR*, *HIF1A*, and *VEGFA* as markers of pathways that may be affected indirectly by *BRAF* expression [30,31]. The gene expression comparison was performed on the same patients who were selected to be evaluated for MAPK activation. RNA expression was measured in TPM (transcripts per million molecules), a transcript-length-normalized value that provides good quantitative estimates of transcript abundance. The RNASeq pipeline does not distinguish between V600E or WT transcripts, so the expression should be considered a sum of all near-wild-type fragments that had paired read data TPMs are log_10_ normal; all boxplots and *t*-tests utilize the log_10_ transformed data. No normalizations were performed. The statistical test used was Welch’s *t*-test for unequal variances and *p*-values were not corrected for multiple testing. For OS, a two-tail *t*-test for groups with unequal variances was used (heteroscedastic).

## 5. Conclusions

In conclusion, there is an observed difference in therapeutic response to BRAF inhibitors in patients with different cancers harboring *BRAF* V600E mutations. Our work suggests that, at least in part, therapeutic response may be explained by the expression levels of mRNA for *BRAF* and the degree of activation of the MAPK pathway and resistance genes in the presence of the *BRAF* V600E mutant, and that this may be context-dependent based on the type of cancer. It might be possible, with this type of information, to select which patients with advanced papillary thyroid cancer or melanoma might benefit most from treatment with BRAF inhibition. Genomic characterization with full annotation to treatment and response may better elucidate the best treatments for these patients.

## Figures and Tables

**Table 1:** The number of patients in our database with melanoma and thyroid cancer cases stratified by their *BRAF* mutation status.

Group	Melanoma	Thyroid Cancer
*BRAF* WT	134	29
*BRAF* V600E Mutation	33	19
Other *BRAF* V600 Mutations	14	0
Miscellaneous *BRAF* mutations	19	1

**Table 2:** Demographics for the cohort of patients. Please note that only papillary thyroid cancer is included in the analysis.

Patient	Age at Diagnosis	Gender	Diagnosis	Stage	BRAF V600E Y/N
1	60	Male	Medullary thyroid carcinoma	3	N
2	36	Female	Papillary thyroid carcinoma	1	Y
3	68	Female	Papillary thyroid carcinoma	2	Y
4	36	Female	Papillary thyroid carcinoma	1	N
5	69	Female	Follicular thyroid carcinoma	1	N
6	51	Male	Papillary thyroid carcinoma	1	Y
7	43	Male	Medullary thyroid carcinoma	4A	N
8	37	Female	Papillary thyroid carcinoma	1	N
9	62	Male	Papillary thyroid carcinoma	2	Y
10	50	Male	Papillary thyroid carcinoma	1	Y
11	35	Female	Papillary thyroid carcinoma	1	Y
12	51	Male	Papillary thyroid carcinoma	4	N
13	44	Male	Papillary thyroid carcinoma	4	N
14	46	Male	Papillary thyroid carcinoma	2	Y
15	60	Female	Medullary thyroid carcinoma	2A	Y
16	62	Female	Papillary thyroid carcinoma	2	Y
17	62	Female	Follicular thyroid carcinoma	1	N
18	55	Male	Papillary thyroid carcinoma	4B	Y
19	52	Male	Papillary thyroid carcinoma	1	Y
20	59	Male	Papillary thyroid carcinoma	2	N
21	47	Male	Follicular thyroid carcinoma	1	N
22	49	Male	Papillary thyroid carcinoma	2	Y
23	41	Male	Papillary thyroid carcinoma	1	N
24	55	Female	Papillary thyroid carcinoma	2	N
25	21	Female	Papillary thyroid carcinoma	1	Y
26	40	Female	Follicular thyroid carcinoma	1	N
27	23	Female	Papillary thyroid carcinoma	2	Y
28	81	Male	Anaplastic thyroid carcinoma	4B	N
29	79	Male	Anaplastic thyroid carcinoma	4A	N

**Table 3:** Demographics for cohort of patients included in the analysis with melanoma.

Patient	Age at Diagnosis	Gender	Stage	BRAF V600E Y/N
1	48	Female	4	Y
2	43	Female	4	Y
3	59	Male	4	N
4	21	Male	1B	Y
5	79	Female	4	N
6	57	Male	4	Y
7	54	Male	4	N
8	50	Female	4	N
9	58	Female	4	N
10	37	Female	4	Y
11	67	Male	4	N
12	64	Male	3B	N
13	77	Male	3C	N
14	65	Male	3C	Y
15	53	Female	3B	Y
16	72	Female	4	N
17	83	Male	4	N
18	47	Male	3	N
19	71	Male	4	N
20	88	Female	3C	N
21	34	Female	4	Y
22	63	Male	4	Y
23	49	Male	4	Y
24	39	Female	3C	Y
25	64	Male	3C	Y
26	55	Female	4	Y
27	66	Male	3	Y
28	78	Male	4	N
29	83	Male	3C	Y

## Data Availability

The data that was analyzed in the current study will be available upon reasonable request from the corresponding author.
